# Comparative Assessment of Azithromycin and Erythromycin for Identifying Inducible Clindamycin Resistance in Staphylococcus aureus

**DOI:** 10.7759/cureus.90166

**Published:** 2025-08-15

**Authors:** Archana LNU, Pooja Kumar, Anupama Singh, Mukesh Kumar, Kumar Abhishek

**Affiliations:** 1 Microbiology, Netaji Subhas Medical College and Hospital, Bihta, Patna, IND; 2 Microbiology, Employees' State Insurance Corporation Medical College and Hospital, Bihta, Patna, IND; 3 Physiology, Indira Gandhi Institute of Medical Sciences, Patna, IND

**Keywords:** azithromycin, d test, erythromycin, icr–inducible clindamycin resistance, staphylococcus aureus

## Abstract

Background

*Staphylococcus aureus* remains a significant pathogen responsible for a wide range of infections. Clindamycin is frequently employed as an effective alternative for treating Staphylococcal infections. However, the emergence of antimicrobial resistance, particularly inducible clindamycin resistance (ICR), poses a substantial therapeutic challenge. Routine detection of ICR is crucial to prevent therapeutic failures and ensure appropriate use of clindamycin. Identifying a suitable substitute for erythromycin in resource-limited settings could enhance the reliability and feasibility of ICR detection in clinical laboratories. This study aimed to assess whether azithromycin could serve as a reliable alternative inducer for the detection of ICR in *S. aureus* and to compare its results with the standard erythromycin-induced D-test.

Material and methods

A cross-sectional comparative study was carried out in a tertiary care hospital setting. A total of 216 non-duplicate clinical isolates of *Staphylococcus aureus* were subjected to the D-test using both erythromycin and azithromycin in combination with clindamycin. The erythromycin-induced clindamycin resistance test, as recommended by the Clinical and Laboratory Standards Institute (CLSI), served as the reference method. Interpretation of azithromycin-induced D-test results was based on adapted CLSI criteria used for erythromycin.

Result

Of the 216 *Staphylococcus aureus* isolates tested, the iMLSB phenotype was most common with 52.31% isolates (n=113), followed by 12.03% cMLSB (n=26) and macrolide-streptogramin (MS) (17.21%, n=37). Our findings show 100% agreement between the test method (using azithromycin) and the reference method (using erythromycin) for the determination of ICR.

Conclusion

This study demonstrates that azithromycin may be a viable alternative to erythromycin for detecting inducible clindamycin resistance in clinical isolates of *Staphylococcus aureus*. Incorporating azithromycin in routine susceptibility testing could offer a practical substitute, particularly in settings where erythromycin is unavailable.

## Introduction

*Staphylococcus aureus* is a significant human pathogen responsible for a broad spectrum of diseases, ranging from minor skin infections to severe systemic conditions such as pneumonia, bacteremia, and endocarditis [1]. The rising incidence of antimicrobial resistance in *S. aureus* strains [2] presents a major therapeutic challenge, complicating the effective treatment of various infections. Methicillin-resistant *S. aureus* (MRSA) prevalence demonstrates geographic variation, with India reporting 44.5% according to Indian Council of Medical Research (ICMR) data [3]. In contrast, Nigeria reported 28.7% for community-acquired and 55.7% for hospital-acquired MRSA [4]. Clindamycin, a lincosamide antibiotic, is frequently used as an alternative therapeutic agent for staphylococcal infections, particularly in cases involving methicillin-resistant *S. aureus* (MRSA) or in patients with β-lactam allergies [5]. Clindamycin resistance among *S. aureus* isolates shows regional variability, with a prevalence of 26.4% reported in India [3], compared to 68% in São Paulo, Brazil [6], and 32.7% in a multi-centric study from China [7]. However, resistance to clindamycin may occur either constitutively or be inducible, necessitating the use of accurate phenotypic detection methods in clinical laboratories [8].

The D-test, a disk diffusion assay, is routinely employed to detect resistance mediated by the macrolide-lincosamide-streptogramin B (MLSB) mechanism. This test allows for the identification of both constitutive (cMLSB) and inducible (iMLSB) resistance phenotypes, as well as the macrolide-streptogramin B (MSB) phenotype in *S. aureus* isolates [9]. Because macrolides and lincosamides share a common ribosomal target site, there is a considerable risk of cross-resistance due to target site modification. Inducible clindamycin resistance in *S. aureus* is primarily mediated by erythromycin ribosome methylase(*erm*) genes, such as *ermA, ermB, and ermC*. These genes encode methyltransferases that methylate the 23S rRNA of the 50S ribosomal subunit, thereby preventing binding of macrolides, lincosamides, and streptogramin B (MLSB) antibiotics [10]. In the inducible phenotype (iMLSB), the expression of *erm *genes is triggered only in the presence of macrolides like erythromycin, but not by clindamycin alone. As a result, isolates may appear susceptible to clindamycin in routine testing, but resistance is expressed when exposed to macrolides -- posing a risk of clinical treatment failure. Recognizing the clinical implications of inducible resistance, the Clinical and Laboratory Standards Institute (CLSI) recommends routine screening for inducible clindamycin resistance (ICR) in all *S. aureus* isolates [11]. ICR refers to the phenomenon wherein bacterial isolates, including *S. aureus* and *Streptococcus *spp., exhibit apparent susceptibility to clindamycin in vitro but can develop resistance when exposed to macrolides such as erythromycin or azithromycin. This inducible mechanism often results in therapeutic failure if clindamycin is used without prior detection of the resistance phenotype [12].

In this context, the present study was undertaken to evaluate the prevalence of inducible clindamycin resistance in clinical isolates of *Staphylococcus aureus*. Furthermore, it aims to compare the performance of erythromycin and azithromycin as inducers of clindamycin resistance. Azithromycin was explored as an alternative to erythromycin in this study due to occasional supply shortages of erythromycin discs, likely linked to its extensive use across standard antimicrobial susceptibility testing (AST) panels for multiple organisms (e.g., *Enterococcus*). Additionally, azithromycin's broader clinical usage and the declining use of erythromycin in current therapeutic settings suggest that azithromycin may better reproduce in vitro the inducible resistance patterns reflective of actual clinical exposures. There is also a theoretical rationale that azithromycin may serve as a more potent or sensitive inducer of *erm*-mediated methylase expression, warranting its evaluation in inducible clindamycin resistance detection.

## Materials and methods

This cross-sectional comparative study was conducted in the Department of Microbiology at a tertiary care hospital in Bihar over a period of one year. Ethical clearance for the study was obtained from the Clinical Research Ethical Committee under approval number CREC/2025/94. As the study was conducted on anonymized stored bacterial isolates obtained through standard diagnostic procedures and did not involve human participants directly, the requirement for patient consent was waived.

Sample size calculation

The sample size was calculated using the single proportion formula, n=Z2p (1-p)/d2. Z is the Z-score corresponding to a 95% confidence interval (1.96), p is the expected proportion, and d is the desired margin of error (0.05). Assuming an expected 16% prevalence of inducible clindamycin resistance among *Staphylococcus aureus* isolates [13], a 95% confidence level, and an absolute precision of 5%, the minimum required sample size was 207. All non-duplicate *S. aureus* isolates recovered from diverse clinical specimens (constituted in decreasing order by pus swab, pus aspirate, urine, and blood) submitted to the microbiology laboratory during the study period were included. Isolates of other bacterial species were excluded.

Procedure and data collection

A total of 216 non-duplicate *S. aureus* isolates were evaluated for inducible clindamycin resistance using both erythromycin and azithromycin as potential inducers. The CLSI-recommended D-zone test employing erythromycin and clindamycin discs served as the reference method. For assessment of azithromycin-induced clindamycin resistance, the same interpretative criteria as those defined by CLSI M100 for erythromycin-induced resistance were applied [11].

**Figure 1:**
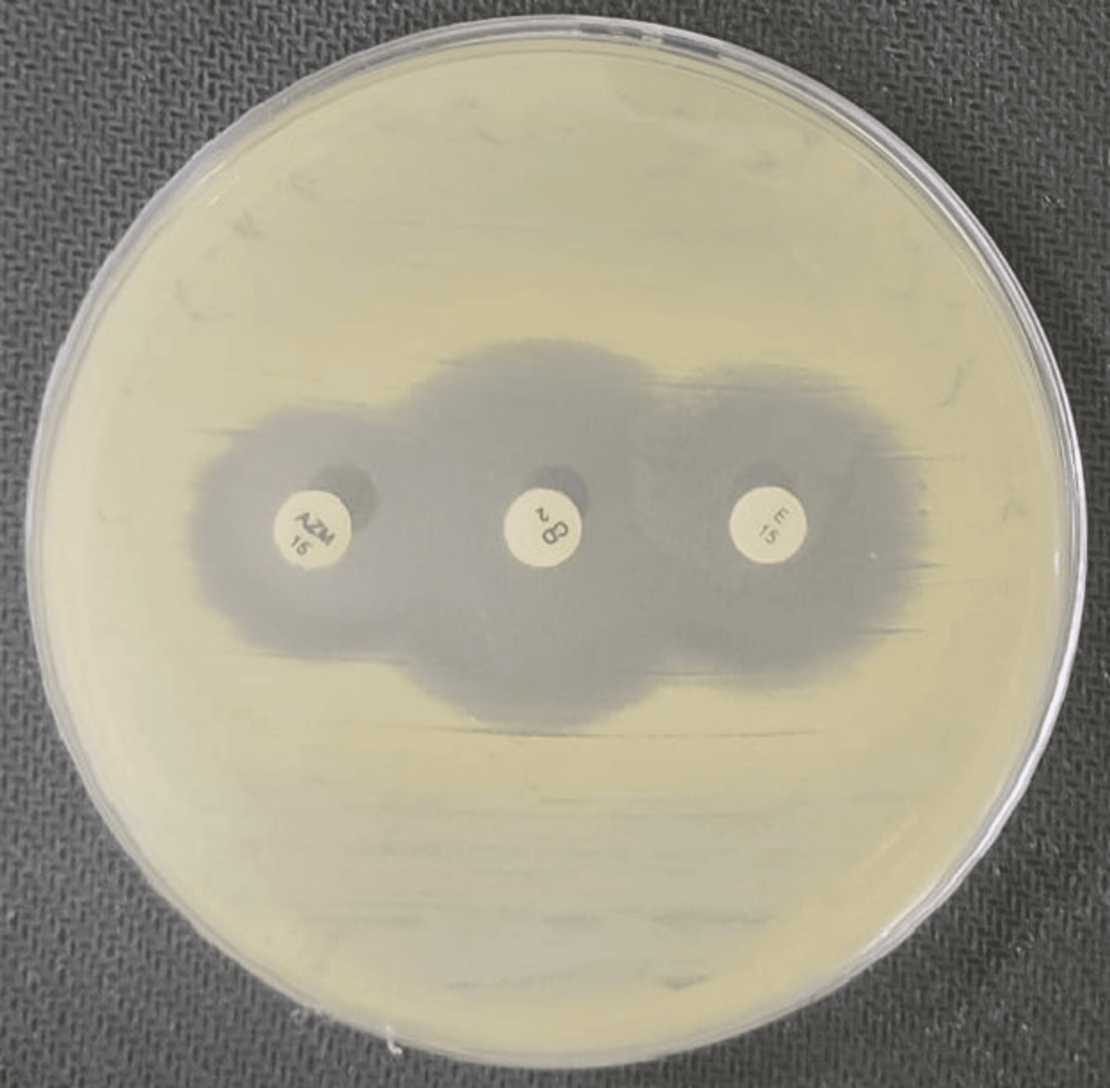
Showing placement of clindamycin, erythromycin, and azithromycin antibiotic discs forming a straight line.

To evaluate the agreement between reference method and test method for inducible clindamycin resistance in *S. aureus* isolates, erythromycin (15 µg) and azithromycin (15 µg) discs were each positioned 15-26 mm apart (edge to edge) from a central clindamycin disc (2 µg) on Mueller-Hinton agar (MHA) plates inoculated with test isolates. The discs were arranged such that the clindamycin disc was equidistant from both erythromycin and azithromycin discs, forming a straight line as shown in Figure 1. This placement ensured uniform exposure of the bacterial lawn to both potential inducers, minimizing spatial bias. To reduce observer bias, all D-zone test results were interpreted independently by more than two microbiologists.

All antibiotic discs were procured from HiMedia Laboratories (India). Plates were incubated in ambient air at 35 ± 2°C for 16-18 hours, and zone diameters were measured according to the CLSI M100 guidelines [11].

Phenotypic classification of resistance

Phenotypically, the clindamycin resistance was defined as: 1. Constitutive-resistant phenotypes (cMLSB): Isolates resistant to both erythromycin (zone diameter ≤13 mm) and clindamycin (≤14 mm). 2. Inducible-resistant phenotypes (iMLSB): Isolates resistant to erythromycin and susceptible or intermediate to clindamycin (zone ≥15 mm), with a characteristic D-shaped blunting of the clindamycin inhibition zone adjacent to the erythromycin disc, such that the zone flattening is towards the erythromycin disk. 3. MS phenotype: Isolates resistant to erythromycin but susceptible to clindamycin without evidence of D-zone formation.

Data analysis

Microsoft Excel (Microsoft Corp., Redmond, Washington) was used to enter all of the data, and SPSS version 20 (IBM Corp., Armonk, NY) was used for analysis. Frequencies and counts were employed for descriptive analysis.

## Results

A total of 216 *S. aureus* isolates during the study period were subjected to phenotypic detection of clindamycin resistance using both reference and test methods, as shown in Table 1.

**Table 1:** Phenotypic patterns and reference method concordance for Staphylococcus aureus isolates. *Based on antibiotic susceptibility to clindamycin and erythromycin. **ER-S: erythromycin susceptible, CL-S: clindamycin susceptible, ER-R: erythromycin resistant, CL-R: clindamycin-resistant, MRSA: methicillin-resistant *S. aureus.*

Phenotype*	ER/CL susceptibility pattern**	Total no. of *Staphylococcus aureus* (%)	No. of MSSA (%)	No. of MRSA (%)	Concordance with reference method (%)
L phenotype	ER-S/CL-R	1 (0.46)	0 (0.00)	1 (0.46)	1/1 (100)
cMLSB phenotype	ER-R/CL-R	26 (12.03)	5 (2.31)	21 (9.72)	26/26 (100)
iMLSB phenotype	ER-R/CL-S with D-zone	113 (52.31)	21 (9.72)	92 (42.59)	113/113 (100)
MS phenotype	ER-R/CL-S without D-zone	37 (17.13)	7 (3.24)	30 (13.89)	37/37 (100)
Susceptible phenotype	ER-S/CL-S	39 (18.06)	21 (9.72)	18 (8.33)	39/39 (100)

**Figure 2:**
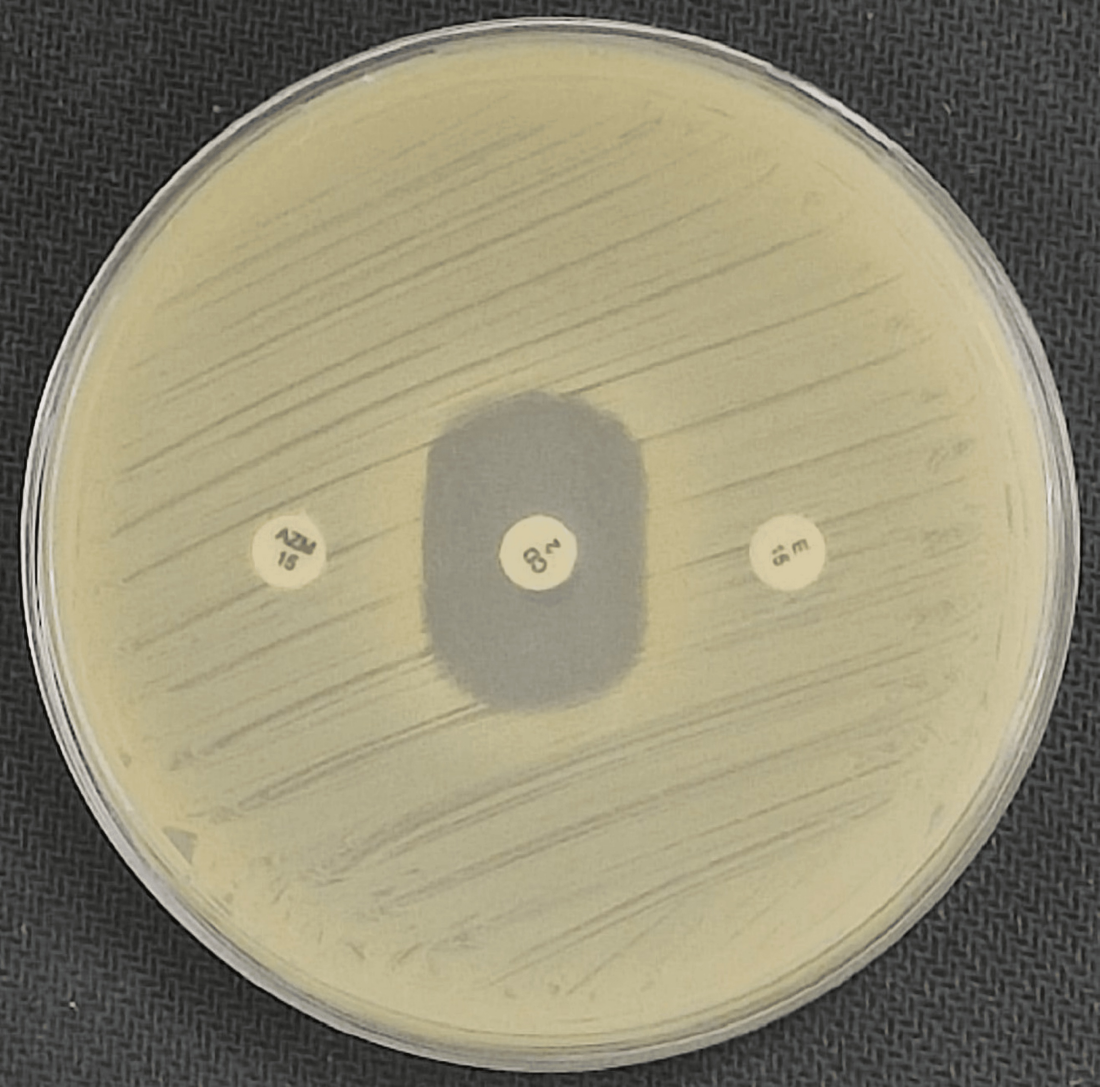
Showing iMLSB phenotype (inducible clindamycin resistance) with both erythromycin and azithromycin.

**Figure 3:**
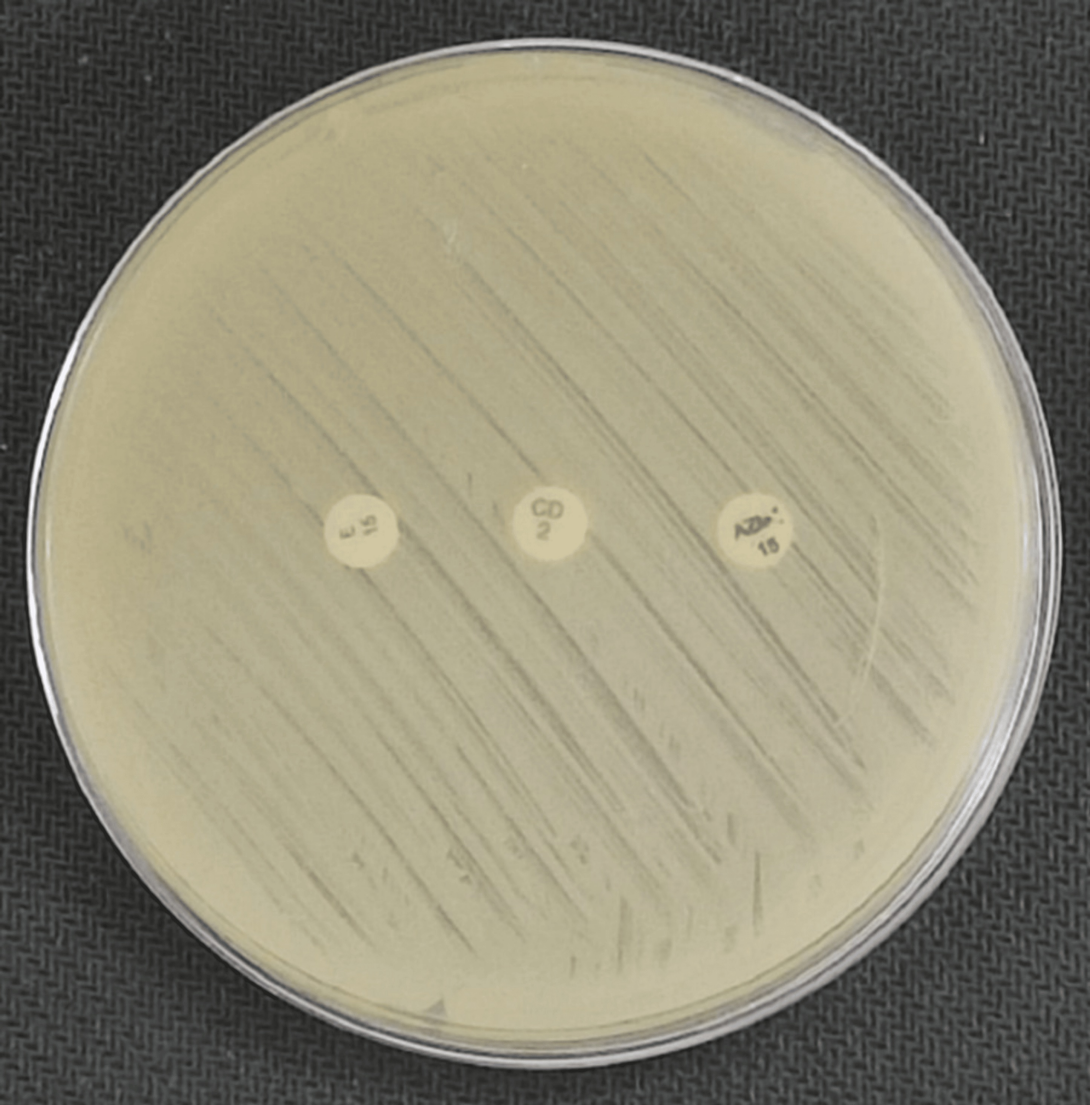
Showing cMLSB phenotype (constitutive resistance) with both erythromycin and azithromycin.

**Figure 4:**
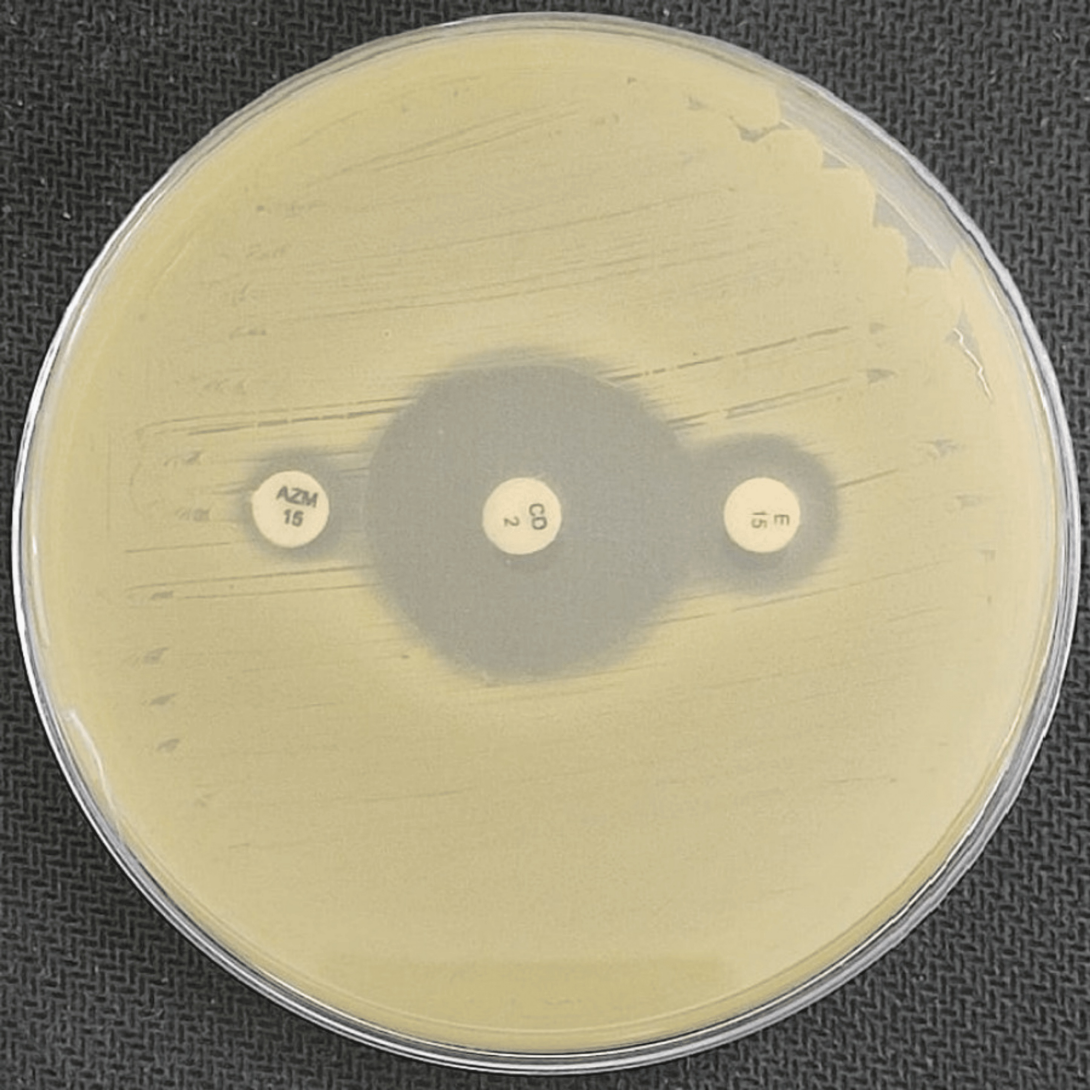
Showing MS phenotype (clindamycin susceptible and erythromycin-azithromycin resistant). MS: macrolide-streptogramin.

Among these, the iMLSB phenotype (inducible resistance) was the most prevalent, observed in 113 isolates (52.31%), with 100% concordance (113/113) between the test and reference methods. This group included 92 MRSA (81.42%) and 21 MSSA (18.58%) isolates. The cMLSB (constitutive resistance) was detected in 26 isolates (12.03%), comprising 21 MRSA and five MSSA, with a 100% concordance rate (26/26). The MS phenotype (erythromycin-resistant, clindamycin-susceptible) was identified in 37 isolates (17.21%), again showing complete 100% agreement (37/37) between both methods. The demonstration of these phenotypes is shown in Figures 2-4, respectively.

**Figure 5:**
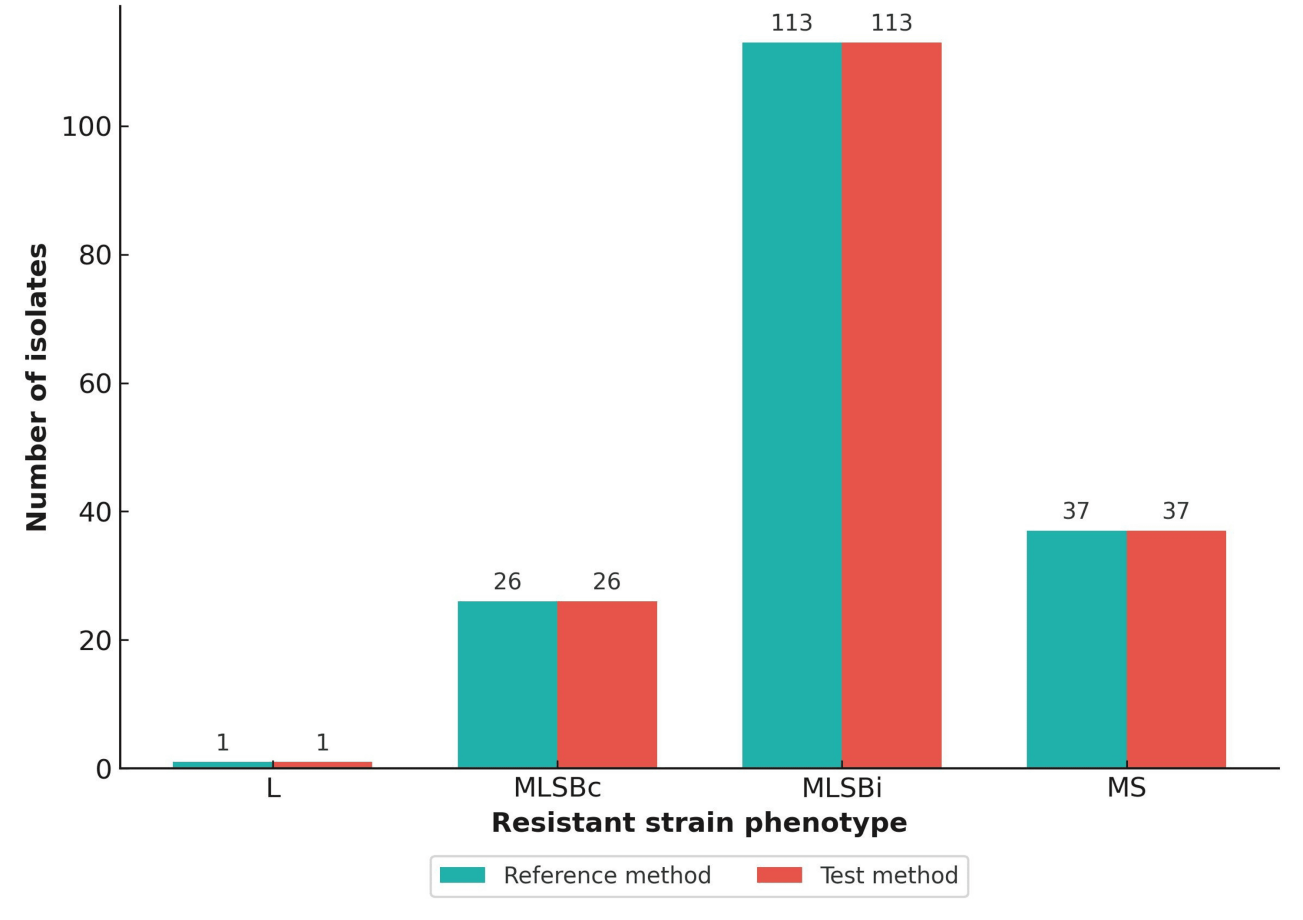
Grouped bar chart showing comparison of resistant strain phenotypes detected by the reference method and the test method. MLSB: macrolide-lincosamide-streptogramin B.

Additionally, 39 isolates (18.05%) demonstrated susceptibility to both erythromycin and clindamycin (as previously shown in Figure 1), all showing perfect agreement (39/39). L phenotype (clindamycin-resistant, erythromycin-susceptible) was seen in a single isolate (0.46%), which was also consistently identified by both methods (1/1). These findings show excellent agreement between the test and reference methods across all clindamycin resistance phenotypes (as depicted in Figure 5). All erythromycin-susceptible strains were also susceptible to azithromycin. Statistical analysis demonstrated perfect agreement between the test and reference methods, with a Cohen's Kappa value of 1.0, indicating complete concordance. Performance metrics, including accuracy, sensitivity, and specificity, were all 100%, confirming the reliability of the test method in detecting inducible clindamycin resistance.

## Discussion

Inducible clindamycin resistance in *Staphylococcus aureus* is a clinically significant phenomenon, as its presence can lead to therapeutic failures if undetected. Identifying this resistance is crucial for guiding appropriate antibiotic therapy and avoiding ineffective treatment regimens [12].

In our study, the prevalence of inducible clindamycin resistance, as determined by the D-test, was 52.31% among *S. aureus* isolates. This is notably higher than rates reported in other regions of India, highlighting substantial geographical variability. For instance, a study by Goyal and Singh [13] in North India reported a much lower prevalence of 15.8%, Majhi et al. [14] in Eastern India observed a 22% D-test positivity rate, while Prabhu et al. [15] reported a 10% incidence. Similarly, Timsina et al. have reported a 23.4% of iMLSB in Nepal [16]. Additionally, Majhi et al. found 19.6% of isolates displaying the MS phenotype (D-test negative), which is comparable to our rate of 17.21%, and 17.7% with constitutive MLSB (cMLSB) resistance, which exceeds our observed rate of 12.03%. Such differences may reflect variations in antibiotic usage patterns, infection control practices, or strain-specific resistance mechanisms. Our findings of iMLSB (inducible clindamycin resistance) far exceed those of the studies done by Majhi et al. and Saxena et al. [14,17].

A key finding of our study is the 100% concordance between erythromycin and azithromycin in detecting inducible clindamycin resistance using the D-test. This indicates that azithromycin can be reliably used in place of erythromycin for identifying iMLSB phenotypes. Our results are consistent with those of Goyal and Singh [13] and Azap et al. [18], who also reported complete agreement between erythromycin and azithromycin for this purpose.

Azithromycin, a first-generation azalide derived from erythromycin, offers several pharmacokinetic and clinical advantages. Its dibasic structure allows for better tissue penetration, prolonged half-life, and reduced dosing frequency [19,20]. These characteristics enhance its appeal for clinical use. However, its broader spectrum and prolonged presence in tissues may also contribute to a higher potential for resistance induction compared to other macrolides like erythromycin and clarithromycin [14,21].

While our findings support azithromycin as a valid alternative for detecting inducible clindamycin resistance, caution is warranted regarding its use in treatment. Azithromycin's stronger propensity to select for resistant strains necessitates careful antibiotic stewardship, particularly in settings with high resistance rates. This is especially relevant in invasive *S. aureus* infections, where even a minor risk of treatment failure should discourage the use of clindamycin without prior resistance testing.

Ultimately, the D-test remains a simple, cost-effective, and essential tool in clinical microbiology for detecting inducible clindamycin resistance. Our results underscore the importance of incorporating this test into routine laboratory workflows, especially given the rising global incidence of macrolide resistance. Clinicians should rely on accurate susceptibility testing to guide therapy and avoid contributing to the growing problem of antimicrobial resistance.

Limitations and scope for further research

Single-center study may restrict the generalizability of the results to other regions or healthcare settings with different bacterial resistance profiles. Future research could adopt a multi-center design with a larger and diverse sample size to enhance external validity.

While statistically adequate, the number of *Staphylococcus aureus* isolates analyzed could be expanded in future studies to strengthen the reliability and applicability of the results.

The current analysis focused solely on *S. aureus* isolates. Inclusion of other clinically significant Gram-positive organisms -- particularly *Streptococcus *species -- would reduce selection bias and provide a more comprehensive understanding of inducible clindamycin resistance patterns across pathogens.

## Conclusions

This study confirms that azithromycin is a reliable alternative to erythromycin for detecting inducible clindamycin resistance in *Staphylococcus aureus* using the D-test, demonstrating 100% concordance across all resistance phenotypes. Incorporating azithromycin into routine susceptibility testing can be particularly valuable in laboratories where erythromycin is unavailable or impractical to use. However, ongoing surveillance and larger multi-centric studies are needed to validate these findings across diverse geographical regions and clinical settings. Further research should also explore the molecular confirmation of inducible clindamycin resistance involving the detection of *erm *genes-primarily *erm*(*A*)*, erm*(*B*)*, and erm*(*C*)-using polymerase chain reaction (PCR)-based methods, which provide definitive evidence of the underlying resistance mechanism.
